# P-262. Prevalence and screening outcomes for human immunodeficiency virus in a rural Appalachian health system hospital

**DOI:** 10.1093/ofid/ofaf695.483

**Published:** 2026-01-11

**Authors:** Caitlin M Guerin, Mathew Watts, Jacob Johnson, Lindsey Sheehan, Christian Rhudy, Daniel Moore

**Affiliations:** University of Kentucky St. Claire, Jeffersonville, KY; University of Kentucky St. Claire, Jeffersonville, KY; University of Kentucky, Lexington, Kentucky; University of Kentucky HealthCare, Lexington, Kentucky; University of Kentucky HealthCare, Lexington, Kentucky; University of Kentucky, Lexington, Kentucky

## Abstract

**Background:**

Despite wide availability of human immunodeficiency virus (HIV) treatment, significant barriers exist in screening and continuity of care. In 2022, the rate of HIV in Kentucky was 10.6 per 100,000 people, however, underscreening in rural populations (especially in Appalachia) may contribute to underreporting of rates. Patients in rural settings are likely to use emergency department (ED) resources, therefore, screenings in this setting may be effective. This study aimed to evaluate the prevalence of HIV as well as coinfection rates with hepatitis B virus (HBV) and hepatitis C virus (HCV) in a rural Appalachian ED.

**Figure:**
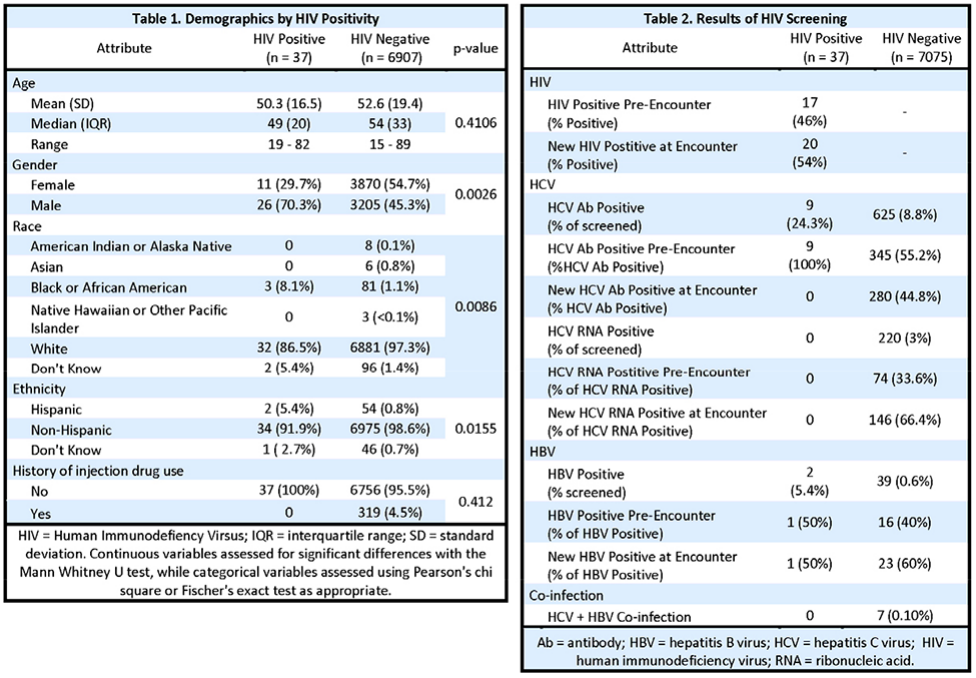

**Methods:**

This retrospective, observational study examined the prevalence of HIV in patients screened in the UK St. Claire ED. A non-targeted screening protocol for HIV, HCV, and HBV was implemented to screen all patients undergoing laboratory studies. Screenings were not performed in patients declining consent or at provider discretion. All patients screened at the UK St. Claire ED between 08/01/24 - 03/31/25 were included. Demographic data and laboratory results were collected from the electronic medical record. Significant differences in patients with HIV positivity were assessed with the Mann Whitney U test, Pearson’s chi square, or Fisher’s exact test as appropriate. HIV, HBV, and HCV screening and positivity are reported in aggregate and positives were classified as either previously diagnosed or newly identified.

**Results:**

7,112 patients were seen in the ED and screened for HIV in the 8-month timeframe. 37 were HIV positive, including 20 (54%) newly diagnosed, indicating a rate of 520 diagnoses per 100,000 patients. Patients with HIV positivity were more frequently male (n=26, 70.3%; negative n=3,205, 45.3%; p=0.0026), and higher proportions were Black or African American (n=3, 8.1%; negative n=81, 1.1%; p=0.0086) and/or Hispanic (n=2, 5.4%; negative n=54, 0.8%, p=0.0155). HCV was observed in 220 patients (3%) and HBV in 39 (0.6%). 2 patients had HIV/HBV, and no HIV/HCV coinfection was observed.

**Conclusion:**

The observed HIV positivity rates at this rural Appalachian ED were high compared to reported state averages. Population composition differed from previously observed trends. Programs to increase screening rates in the Appalachian region are needed.

**Disclosures:**

Caitlin M. Guerin, PharmD, Gilead: Grant/Research Support Christian Rhudy, PharmD, MBA, Gilead Sciences, Inc.: Grant/Research Support Daniel Moore, MD, MBA, FACEP, Gilead Sciences: Grant/Research Support

